# A Novel Nanosystem Realizing Curcumin Delivery Based on Fe_3_O_4_@Carbon Dots Nanocomposite for Alzheimer’s Disease Therapy

**DOI:** 10.3389/fbioe.2020.614906

**Published:** 2020-12-03

**Authors:** Ying Kuang, Jingwen Zhang, Mogao Xiong, Weijia Zeng, Xiaofeng Lin, Xiaoqing Yi, Yan Luo, Min Yang, Feng Li, Qitong Huang

**Affiliations:** ^1^Guangdong Provincial Key Laboratory of Brain Function and Disease, Department of Anatomy and Neurobiology, Zhongshan School of Medicine, Sun Yat-sen University, Guangzhou, China; ^2^Oil-tea in Medical Health Care and Functional Product Development Engineering Research Center in Jiangxi, Key Laboratory of Biomaterials and Biofabrication in Tissue Engineering of Jiangxi Province, Key Laboratory of Prevention and Treatment of Cardiovascular and Cerebrovascular Diseases, Ministry of Education, Gannan Medical University, Ganzhou, China; ^3^Department of Chemistry, Shantou University, Shantou, China

**Keywords:** curcumin, Alzheimer’s disease, Aβ protein, carbon dots, Fe_3_O_4_ nanomaterial, drug delivery

## Abstract

Alzheimer’s disease (AD) is the most common neurodegenerative disease, which seriously affects human health but lacks effective treatment methods. Amyloid β (Aβ) aggregates are considered a possible target for AD treatment. Evidence is increasingly showing that curcumin (CUR) can partly protect cells from Aβ-mediated neurotoxicity by inhibiting Aβ aggregation. However, the efficiency of targeted cellular uptake and bioavailability of CUR is very low due to its poor stability and water-solubility. In order to better improve the cell uptake efficiency and bioavailability of CUR and reduce the cytotoxicity of high-dose CUR, a novel CUR delivery system for AD therapy has been constructed based on the employment of the Fe_3_O_4_@carbon dots nanocomposite (Fe_3_O_4_@CDs) as the carrier. CUR-Fe_3_O_4_@CDs have a strong affinity toward Aβ and effectively inhibit extracellular Aβ fibrillation. In addition, CUR-Fe_3_O_4_@CDs can inhibit the production of reactive oxygen species (ROS) mediated by Aβ fibrils and the corresponding neurotoxicity in PC12 cells. More importantly, it can restore nerve damage and maintained neuronal morphology. These results indicate that the application of CUR-Fe_3_O_4_@CDs provides a promising platform for the treatment of AD.

## Introduction

Alzheimer’s disease (AD) is a neurodegenerative disease characterized by progressive cognitive and memory impairment, which has become one of the great challenges of health care in the 21st century. There are little approved treatments can reverse or prevent the development of this disease (Scheltens et al., 2016; Hodson, 2018; Long and Holtzman, 2019). The pathological feature of AD is abnormal folding and deposition of amyloid plaques (Amyloid β, Aβ) leads to formation of senile plaque (SP), and the accumulation of tau protein leads to neurofibrillary tangles (NFT) (Simard et al., 2006; Bulgart et al., 2020). Aβ_42_ fibrils is a major component of amyloid plaques and appears central to AD pathogenesis (Fagan et al., 2006). The neurofibrillary tangle of tau protein is thought to be caused by the imbalance between Aβ production and Aβ clearance (Hardy and Higgins, 1992; Iadanza et al., 2018; Grasso et al., 2019; van der Kant et al., 2020). Therefore, the development of AD treatment mainly focuses on the removal of Aβ from the brain (Saravanan et al., 2020). The aggregation of Aβ in the brain induces immune-inflammatory response and neurotoxicity, eventually leading to the occurrence and development of AD, which presents typical pathological changes and clinical symptoms (Jaunmuktane et al., 2015).

Curcumin (CUR) is a small polyphenol molecule extracted from *Curcuma longa L*, which is generally considered to be the most effective ingredient (Wang et al., 2020). The CUR has anti-cancer, anti-inflammatory, anti-oxidant, anti-bacterial, and neuroprotective effects, and is a very potential drug for the prevention or treatment of AD (Esatbeyoglu et al., 2012). Extensive data from *in vitro* studies showed that CUR inhibited the formation of Aβ oligomers and Aβ fibrils in a dose-dependent manner (Reinke and Gestwicki, 2007; Reddy et al., 2018). An AD 5 × FAD mouse model experiment showed that CUR can reduce the production of Aβ by down-regulating β-site APP cleaving enzyme 1 (BACE1) expression, preventing synapse degradation, and improving spatial learning and memory disorders (Zheng et al., 2017). However, CUR is photodegradable, can self-degradable in the dark (Mondal et al., 2016). The cell-targeted uptake efficiency and bioavailability of CUR is very low, mainly due to its poor water solubility and aqueous stability. In addition, CUR can cause obvious cytotoxicity at high concentrations (Baum et al., 2008; Ringman et al., 2012; Moradi et al., 2020). Therefore, the construction of an efficient CUR loading and control–release system which can improve stability, solubility, and biocompatibility is very essential to further improve the CUR curative effect on AD.

Till now, the core-shell nanoparticles are achieved by combination of core and shell materials, which have been widely used in the drug delivery due to their superior features, including high specific surface area, nice dispersity, and good chemical (Cheng et al., 2019; He et al., 2019; Yang et al., 2019). The widely used core materials are such as magnetic nanoparticles, metal nanoparticles, silica nanoparticles, etc. However, the core-shell nanoparticles with the magnetic nanoparticles as core especially have attracted tremendous interest owing to its high magnetic saturation for targeted drug delivery (Liu et al., 2019; Song et al., 2020). Cai et al. (2020) have synthesized Fe_3_O_4_@SiO_2_ nanoparticles for the doxorubicin delivery and release. Kim et al. (2020) have successfully prepared Fe_3_O_4_@Void@microporous organic polymer nanoparticles for the delivery of doxorubicin with satisfactory drug loading efficiency and content to targeted tumor cells. Although these core-shell nanoparticles exhibited the satisfactory drug loading capacity and stability and effectively controls the release of drugs, the complex synthesis process and the poor biocompatibility limited their further application in clinical treatment. More importantly, these core-shell nanoparticles have weak inherent fluorescence to indicate the self-monitoring of the drug carrier. Carbon Dots (CDs), with good fluorescence properties, have been used as an effective guarantee for its potential application in the biomedical field in recent years due to their several advantages, such as good biocompatibility, simple synthesis process, and strong inherent fluorescence (Peng et al., 2017; Wang et al., 2018; Zhao et al., 2018; Rahmati and Mozafari, 2019; Xu et al., 2019; Garner et al., 2020). The CDs also have reducibility, which can be applied as reducing agent and stabilizer to prepare Fe_3_O_4_@CDs nanocomposite. What’s more, CDs can be fixed and loaded with CUR by π-π accumulation and hydrogen bonding interaction. Therefore, the Fe_3_O_4_@CDs nanocomposite cannot only provide excellent CUR loading performance but also provide fluorescence tracer function.

Herein, the Fe_3_O_4_@CDs nanocomposite with good biocompatibility has been synthesized by a hydrothermal method, then the Fe_3_O_4_@CDs nanocomposite was used to load CUR (CUR-Fe_3_O_4_@CDs). CUR-Fe_3_O_4_@CDs have self-fluorescence properties and excellent biocompatibility, making it useful as a fluorescent label for the biomedical imaging. What’s more, CUR-Fe_3_O_4_@CDs can inhibit the aggregation of Aβ protein, the inhibition rate is as high as 92.67%, which shows that it has very good potential application value in the treatment of AD. Besides, CUR-Fe_3_O_4_@CDs can reduce the PC12 neurotoxicity and cell internal ROS induced by Aβ fibrils. Therefore, we conclude that the Fe_3_O_4_@CDs nanocomposite has potential for targeted CUR drug delivery for AD therapy (**Scheme 1**).

## Materials and Methods

### Materials and Reagents

Aβ_42_ was obtained from GL Biochem (Aβ, Shanghai, China). CUR, hexafluoroisopropanol (HFIP), 3-(4,5-dimethylthiazol-2-yl)-2,5-diphenyltetrazolium bromide (MTT), and thioflavin T (ThT) were purchased from Sigma-Aldrich (United States). The rat pheochromocytoma (PC12) cell line was obtained from the Cell Bank, Type Culture Collection Committee, Chinese Academy of Sciences (CBTCCCAS). Fetal bovine serum (FBS), Dulbecco’s modified Eagle’s medium (DMEM), and penicillin–streptomycin solution were purchased from Life Technologies Inc. (United States). Dihydroethidium (DHE) was obtained from Jiancheng Bio (China). FeCl_3_, glucose, CH_3_COONa, citric acid, and sodium acetate were purchased from Xilong Chemical Co., Ltd. (China).

Transmission electron microscopy (TEM) was determined by Tecnai G2 F30. X-ray diffraction (XRD) patterns were measured on Bruker D8 Advance. UV-vis absorption was carried out on Specord 50 plus. Cytotoxicity assays were measured on enzyme-labeled instrument (Thermo Varioskan LUX). Cell imaging was determined by Zeiss LSM 880.

### Synthesis of Fe_3_O_4_@CDs Nanocomposite

Synthesis of CDs: 3 g citric acid and 1 g glucose were well mixed to the synthesis of CDs by using a microwave method with the radiation power of 800 W for 5 min. After cooling, the solution was diluted with 30 mL water and filtered by a 0.22 μm microporous membrane; then the above solution was centrifuged at 12000 rpm for 15 min. Finally, the CDs was diluted into a 25 mL aqueous solution.

Synthesis of Fe_3_O_4_@CDs nanocomposite: the as-prepared CDs (15 mL) and 1 g CH_3_COONa were added to 1 g FeCl_3_. The mixed-solution was ultrasonicated for 8 h, then transferred to the hydrothermal reactor and heated at 210°C for 24 h. After cooling, the above solution was centrifuged at 15000 rpm for 10 min to obtain the Fe_3_O_4_@CDs nanocomposite.

### Loading of Fe_3_O_4_@CDs With CUR

Loading of CUR on Fe_3_O_4_@CDs has been performed by mixing CUR (4 mg) and Fe_3_O_4_@CDs (20 mg) in the 30 mL pH = 7.4 phosphate-buffered saline (PBS) buffer solution. After the ultrasonic mixing was uniform, the mixed solution was stirred at 37.0°C for 10 h in the dark condition. The product was then collected by centrifugation and washed with PBS buffer solution three times and freeze-dried. At the same time, the content of CUR in the supernatant was monitored by high-performance liquid chromatography (HPLC). The HPLC conditions were as follows: C18 column (column size: 250 mm × 4.6 mm, particle size: 5 μm) were used. The mobile phase consisted of acetonitrile and 5% acetic acid (75:25, v/v), with a flow rate of 1 mL/min. Chromatography was performed at 30°C and the ultraviolet detection wavelength used was 425 nm. The run time for analysis was 10 min and sample injection volume was 20 μL. The loading efficiency of CUR was calculated as follows:

Drug loading content (wt.%) = (weight of loaded CUR/total weight of Fe_3_O_4_@CDs and loaded CUR) × 100%

Drug loading efficiency (%) = (weight of loaded CUR/weight of drug in feed) × 100%

### *In vitro* Drug Release

The release profiles of CUR from CUR-Fe_3_O_4_@CDs was conducted by a modified dialysis method (Shaikh et al., 2009). A 1.0 mL CUR-CDs and CUR-Fe_3_O_4_@CDs solution (0.4 mg/mL) were added to a dialysis bag (MWCO = 2000 Da), respectively, then immersed in PBS (pH = 5.7 or 7.4) at 37°C with gentle shaking (100 rpm) in the dark. At the predetermined time point (0, 1, 2, 4,8, 12, 18, and 24 h), a 1 mL medium solution was collected and supplemented with an equal amount of fresh medium. The externally visible absorption was measured by HPLC.

### Cytotoxicity

The MTT method was used to monitor the toxicity of CUR-Fe_3_O_4_@CDs to PC12 cells. CUR-CDs and CUR were used as control. PC12 cells were cultured in 96-well plates with a density of 1 × 10^4^ cells/well for 24 h at 37°C and 5% CO_2_. CUR-Fe_3_O_4_@CDs, CUR-CDs, and CUR were diluted into complete DMEM to achieve the desired final concentration and added to the cells, using the cells without added test solution as the positive control and the media without added cells and test solution as blank control. Following treatments, 20 μL MTT (5 mg/mL) was added into each well, and cells were maintained in the incubator for an additional 4 h at 37°C. The supernatant was then aspirated carefully, and 150 μL DMSO was used to dissolve the formazan crystals. The plates were shaken slightly for 10 min to ensure complete dissolution of the formazan crystals. The absorb was measured by Microplate Reader at a wavelength of 490 nm.

### Hemolysis Analyses

Using heparin sodium as an anticoagulant, fresh blood was collected from mice. Blood was centrifuged at 10000 × *g* for 5 min to obtain red blood cells (RBC). After washing three times with PBS, the sample was resuspended with 10% hematocrit (v/v) in PBS. Different concentrations of CUR, CUR-CDs, and CUR-Fe_3_O_4_@CDs in PBS were mixed with 10% hematocrit suspensions, then incubated at 37°C for 3 h. PBS and distilled water were used as negative control and positive control, respectively. After incubation, the supernatant was centrifuged at 10000 × *g* for 5 min. The absorbance of the supernatant at 545 nm was measured using a microplate reader to calculate the percent hemolysis. The formula for calculating the hemolysis rate is (*A*_sample_-*A*_negative_)/(*A*_positive_-*A*_negative_) × 100%, in which *A*_sample_ is the absorbance of the supernatant of red blood cells in the sample group; *A*_positive_ and *A*_negative_ are positive control and negative control, respectively.

### Cellular Uptake of CUR-Fe_3_O_4_@CDs

PC12 cells were seeded on the coverslips in a 24-well plate with the density of 1 × 10^4^ cells/well and cultured for 24 h. CUR-Fe_3_O_4_@CDs was added to the well plate and incubated for 1 h, and the cells were gently washed with PBS three times to remove the attached CUR-Fe_3_O_4_@CDs. Then, the cells were fixed with 4% formaldehyde for 15 min at 4°C, washed by PBS, and the slide sealed with glycerin. Confocal lasing scanning microscopic (CLSM) was used to determine the cell uptake of CUR-Fe_3_O_4_@CDs.

### Molecular Docking

The 2D structure of CUR was obtained from the zinc database and converted into a 3D structure by CORINA Classic. The X-ray structure of the Aβ_42_ protein was downloaded from the RCSB protein database (PDB ID: 1IYT) and the sequence was DAEFRHDSGY10 EVHHQKLVFF20 AEDVGSNKGA30 IIGLMVGGVVIA42 (Tiwari and Kepp, 2015). AutoDock4.2 was used to study the combination of CUR and Aβ_42_. Aβ_42_ remains rigid and CUR is allowed to be flexible. The AutoDock type of CUR is assigned, and 12 active twist angles (rotatable keys) are defined as flexible parts. To explore possible binding sites, the entire Aβ_42_ was used as a blind docking zone. A grid of 76 × 50 × 126 points, with a grid spacing of 0.375 Å, was selected. The grid frame is centered on Aβ_42_β and covers the entire Aβ_42_. Ligand docking was performed using the Lamarck genetic algorithm, with a total of 150 individuals, and 2.5 million energy assessments were performed during 100 runs of 27,000 generations. The docking results were then clustered and the RMS tolerance value was set to 2.0 to determine the main orientation of the ligand.

### Procedure for Aβ_42_ Detection

Thioflavin T is a thiazine dye, which can specifically bind to the β-sheets shared by amyloid protein structure. Thus, the intensity of ThT fluorescence signal can be used to detect the aggregation of amyloid protein directly and quantitatively (Hudson et al., 2009; Wolfe et al., 2010). After treating Aβ_42_ with HFIP, it was dissolved in DMSO (5 mM) and diluted with PBS, then co-incubated with CUR, CUR-CDs, and CUR-Fe_3_O_4_@CDs at 37°C for 0–6 days. The formation of amyloid fibril was detected by ThT binding assay. The co-incubation solution was mixed with ThT, and the fluorescence intensity was tested by multifunctional enzyme marker (Ex 450 nm, Em 482 nm).

### Turbidity Assay

Curcumin, CUR-CDs, and CUR-Fe_3_O_4_@CDs (20 μM) were incubated with Aβ_42_ sample solution at 37°C for 72 h. Then, the sample was mixed with buffer solution (900 μL, 20 mM Tris–HCl, 150 mM NaCl, pH = 7.4), its absorbance measured at 405 nm by ultraviolet-visible absorption spectroscopy, and Aβ_42_ left alone as a control.

### Circular Dichroism (CD) Spectroscopy

Circular dichroism spectra were measured on a BRIGHTTIME spectropolarimeter. CD spectra of Aβ_42_ treated with or without CUR, CUR-CDs, and CUR-Fe_3_O_4_@CDs for 72 h were recorded using a 1 mm path length cell at room temperature in the spectral range 190–290 nm. The scan of the PBS buffer alone was subtracted from the average scan for each sample as the baseline. For each sample, the spectrum was scanned at least three times.

### TEM of Aβ Fibrils With Other Materials

The Aβ solution was incubated for 72 h to obtain abundant Aβ fibrils. The pre-formed Aβ fibrils were incubated with CUR, CUR-CDs, and CUR-Fe_3_O_4_@CDs for 72 h to prepare sample solutions. Each sample was dropped on the carbon-coated copper grid; the excess sample was removed and negatively stained with 2% uranyl acetate for 1 min. The morphology was observed using a TEM.

### Aβ_42_ Toxicity and CUR-Fe_3_O_4_@CDs Treatment

Aβ_42_ was treated with HFIP, dissolved with DMSO, and incubated at 37°C for 6 days to form Aβ_42_ fibrils. PC12 cells were seeded in 96-well plates at a density of 1.0 × 10^4^ cells/well. Cells were treated with freshly prepared concentrations of Aβ_42_ fibrils of different concentrations for 24 h. After standardization of neurotoxicity levels, suitable Aβ_42_ fibrils concentration was used for all experiments with 24 h exposure and with treatment at different concentrations of CUR, CUR-CDs, and CUR-Fe_3_O_4_@CDs. MTT assay was performed as described before.

### Measurement of Reactive Oxygen Species

Dihydroethidium fluorescent probe was used to detect intracellular ROS level. PC12 cells were seeded in a 15-mm glass-bottom dish at a density of 1 × 10^4^ cells/dish for 24 h before experiment. After PC12 cells treating with Aβ_42_ fibrils for 24 h, CUR, CUR-CDs, and CUR-Fe_3_O_4_@CDs were added to intervene for 24 h. Then, the cells were incubated with 10 μM of DHE in the dark at 37°C for 30 min, washing PC12 cells with PBS three times. CLSM was used to image at the excitation wavelength of 518 nm. The image was quantitatively analyzed using Image J software.

### Statistical Analysis

Student’s *t*-test was used for comparing two groups, and one-way ANOVA was used for comparison among multiple groups. All data are presented as means ± S.D. *p* < 0.05 was considered statistically significant.

## Results and Discussion

### Synthesis and Characterization of Fe_3_O_4_@CDs

**FIGURE 1:**
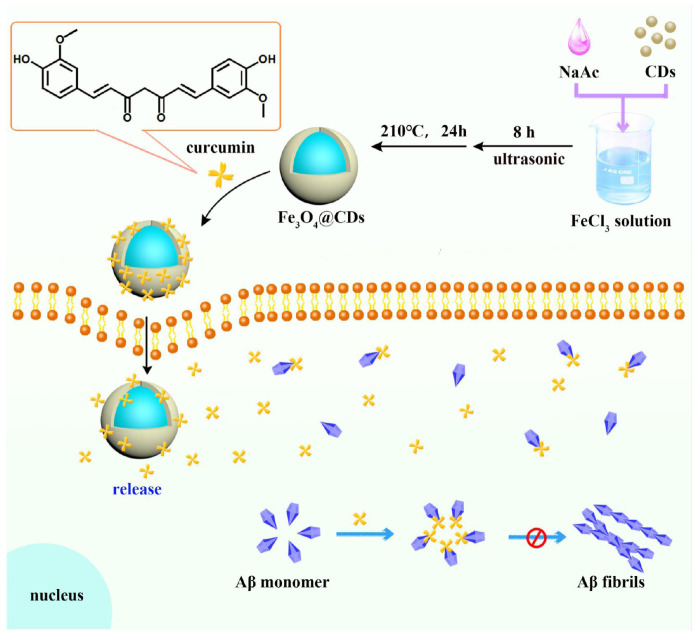
A novel nanosystem realizing curcumin (CUR) loading based on Fe_3_O_4_@CDs nanocomposite for Alzheimer’s disease therapy.

The morphology of CDs and Fe_3_O_4_@CDs was imaged by using TEM. Figure 1A shows the size of CDs is in the range of 0.5–3 nm, From the Figure 1B, we can see that the size of Fe_3_O_4_@CDs is in the range of 5–20 nm. Figure 1C displays an HRTEM image of the sectional hybrid nanoparticles. As shown in Figure 1C, HRTEM image of Fe_3_O_4_@CDs nanocomposite with the lattice spacing around 0.343 nm, corresponding to the (220) plane of the Fe_3_O_4_ (Zhang et al., 2018), and the lattice spacing around 0.211 nm, agreeing well with the crystallographic (002) spacing of carbon (Justin et al., 2016). The results show that the CDs are successfully loaded on the surface of Fe_3_O_4_.

The crystalline structure of the CDs and Fe_3_O_4_@CDs nanocomposite were investigated by X-ray diffraction (XRD) (Figure 1D). The characteristic peaks of CDs appeared at 23.05°, which was indexed to (002) crystal plane (Wang et al., 2017). In a diffraction pattern of the Fe_3_O_4_@CDs nanocomposite, 2θ = 18.23°, 29.98°, 35.29°, 42.91°, 53.18, 56.71, and 62.25° were assigned to the (111), (220), (311), (400), (422), (511), and (440) planes, the results agreed with the standard XRD data for the Fe_3_O_4_ (JCPDS card, No. 76-1849) (Jaiswal et al., 2018). The magnetic property of Fe_3_O_4_@CDs nanoparticle was examined by a VSM at 300K. As shown in Figure 1E, the saturation magnetization value of Fe_3_O_4_@CDs is 18.4 emu/g. The suspension also exhibited a very good magnetic response to an external magnet (Figure 1F), which shows that the Fe_3_O_4_@CDs can target delivery within an external magnetic field. Although the magnetic properties of CUR-Fe_3_O_4_@CDs have been weakened, it still reaches 17.5 emu/g (Figure 1E), indicating that CUR-Fe_3_O_4_@CDs also has very excellent magnetic properties and indirectly indicating that CUR has been successfully loaded on Fe_3_O_4_@CDs. Moreover, UV-Vis absorbance of CDs, Fe_3_O_4_@CDs, and CUR-Fe_3_O_4_@CDs was shown in Supplementary Figure 1. The UV-Vis absorbance of CDs and Fe_3_O_4_@CDs has a significant absorption band at 283 nm, consistent with previous reports (Ponnaiah et al., 2018; Huang et al., 2020). However, when the CUR was loaded on the Fe_3_O_4_@CDs nanoparticle, there is a typical UV-Vis absorption peak at 425 nm, indicating that CUR was loaded on the Fe_3_O_4_@CDs nanoparticle with the load rate was 90.5%. The main reason is that the Fe_3_O_4_@CDs nanoparticle has high surface area and the π-π stacking and hydrogen bonding interaction between the Fe_3_O_4_@CDs and CUR.

### *In vitro* Drug Release

Since nanoscale drug delivery systems only get access to cell through endocytosis, these carriers usually end up in lysosomes, where they are exposed to low pH (usually pH 4.5) and proteolytic enzymes (Xiong et al., 2008). Thus, drug can be triggered form the carrier in lysosomal microenvironment is important. We explore the release rate of CUR from CUR-Fe_3_O_4_@CDs under different pH conditions (pH = 5.7 or 7.4). The accumulative drug release profiles as a function of time are plotted in Supplementary Figure 2, sustainable release of CUR release from CUR-CDs and CUR-Fe_3_O_4_@CDs at pH 5.7 were rapid, and nearly 78.7% and 82.65% of the CUR was released within 24 h, respectively. In contrast, CUR released from CUR-CDs and CUR-Fe_3_O_4_@CDs at pH 7.4 was slower. It shows that acidic conditions are more conducive to the release of CUR. This is because, at a low pH of 5.7, the solubility of CUR can be increased due to the protonation process. The results also indicated that Fe_3_O_4_@CDs could be a good choice for loading CUR and had the ability to enable pH-triggered drug release.

### Biocompatibility Study of CUR-Fe_3_O_4_@CDs

**FIGURE 2:**
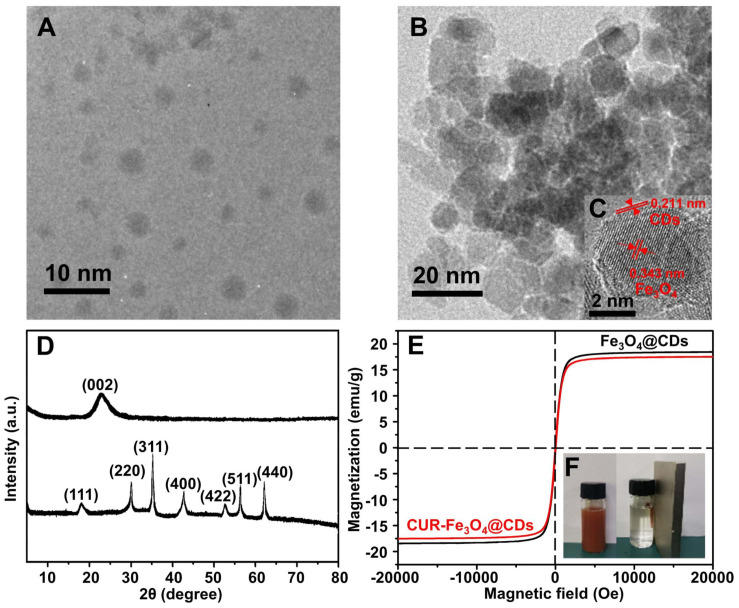
Characterization of nanoparticles. **(A)** TEM image of CDs. **(B)** TEM image of Fe_3_O_4_@CDs. **(C)** HRTEM image of Fe_3_O_4_@CDs. **(D)** XRD spectra of CDs and Fe_3_O_4_@CDs. **(E)** Magnetic hysteresis loops of Fe_3_O_4_@CDs and CUR-Fe_3_O_4_@CDs. **(F)** The photograph of the stable dispersion of Fe_3_O_4_@CDs in water and the corresponding magnetic response of the suspension to a magnet.

The low toxicity and good biocompatibility of nanoparticles is essential to ensure their safe and effective application in AD treatment (Sun et al., 2019). MTT assay was used to investigate the effect of CUR-Fe_3_O_4_@CDs on the survival of PC12 cells. It has been observed that CUR-CDs and CUR-Fe_3_O_4_@CDs showed nearly no cytotoxicity in PC12 cells in a dose-dependent of CUR (5–500 μg/mL) manner, compared to free CUR. The cell viability was 86.44%, 82.36%, and 53.71% in the presence of CUR-Fe_3_O_4_@CDs (equal to 500 μg/mL CUR), CUR-CDs (equal to 500 μg/mL CUR), and CUR for 12 h, respectively. Cell viability was 82.60%, 79.88%, and 33.41% after incubation with materials for 24 h, respectively (Figures 2A,B). These results suggest that CUR-Fe_3_O_4_@CDs and CUR-CDs are much less cytotoxic than CUR. In addition, the biocompatibility of CUR-Fe_3_O_4_@CDs were explored by using RBCs. According to the ASTM F756-17 standard, if the material hemolysis rate is less than 2%, it can be considered to be non-hemolytic. As shown in Figure 2C, hemolysis rate of different concentrations (0.5–50 μg/mL) of CUR were 0.20–2.69%, CUR-CDs were 0.14–0.90%, CUR-Fe_3_O_4_@CDs were 0.11–1.39%. Both CUR-CDs and CUR-Fe_3_O_4_@CDs were less than 2%, no hemolysis occurred. These data indicate that CUR-Fe_3_O_4_@CDs have good blood compatibility.

### Effect of CUR-Fe_3_O_4_@CDs on Aβ_42_ Aggregation

The deposition of Aβ fibrils induces neurotoxicity, oxidative stress, and the loss of synapse, which is the main factor in the pathogenesis of AD (Gandy and DeKosky, 2013). CUR binding to Aβ monomer is essential to inhibit Aβ fibrils formulation and toxicity. Thus, we first studied the binding of CUR and Aβ_42_ monomer. Molecular dynamics calculations were also performed using Autodock4.2 and PyMOL2.3 software to explore the combination of CUR and Aβ_42_. The Supplementary Figure 3 shows the best complex obtained with CUR and monomeric Aβ_42_ peptide. As expected, the ligand is close to the amyloid region corresponding to the aforementioned 16KLVFFA21 sequence, which is well known to participate in ligand recognition. The β-keto-enol central core forms an H bond with ALA-21, while the ligand benzene ring stabilizes the π-π stacking contact with LYS-16. The binding energy is −4.6 cal/mol.

**FIGURE 3:**
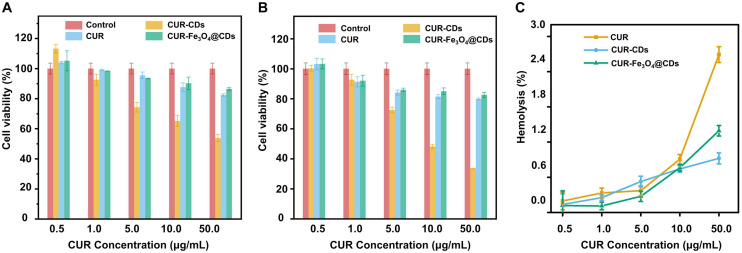
Biocompatibility assay. The cell viabilities of PC12 cells incubated for 12 h **(A)** and 24 h **(B)** with different concentrations of CUR, CUR-CDs, and CUR-Fe_3_O_4_@CDs. **(C)** The hemolysis of CUR, CUR-CDs, and CUR-Fe_3_O_4_@CDs at different concentrations.

In AD, the conformational change of Aβ first changes from random coiling to β-sheet structure, and then Aβ fibril formation occurs (Bleiholder et al., 2011). ThT fluorescence has been widely used to detect the conformation of these β-sheet structures and their Aβ aggregates (Biancalana and Koide, 2010). The fluorescence of ThT was significantly increased in the presence of anti-amyloidogenic compounds by binding to β-sheet of amyloid protein fibril (LeVine, 1993; Biancalana and Koide, 2010). This property of ThT makes it particularly useful to examine the inhibitory effect of our CUR-Fe_3_O_4_@CDs on Aβ fibrillation. ThT fluorescence was used to detect aggregated β-sheet fibrils in the presence of CUR, CUR-CDs, and CUR-Fe_3_O_4_@CDs (1 μg/mL). As shown in Figure 3A, after incubation at 37°C for 6 days, Aβ_42_ produced a typical sigmoid curve, and the fluorescence intensity continued to increase. In addition, after adding CUR, CUR-CDs, and CUR-Fe_3_O_4_@CDs, the increase of fluorescence intensity slowed down. ThT test observed that after 6 days of incubation, the cleansing efficiency of CUR, CUR-CDs, and CUR-Fe_3_O_4_@CDs were up to 66.13%, 88.30%, and 92.67%. In summary, these results show that both CUR-Fe_3_O_4_@CDs and CUR-CDs increase the inhibitory effect of CUR on the aggregation of Aβ_42_ fibrils.

We further studied the inhibitory action of CUR-Fe_3_O_4_@CDs on Aβ aggregation by turbidity method. Turbidity is an important indicator of Aβ_42_ aggregates, reflecting the change of the optical density of Aβ_42_ solution (Wang et al., 2012). As shown in the Figure 3B, only CUR, CUR-CDs, CUR-Fe_3_O_4_@CDs, and Aβ_42_ have very low turbidity values. After Aβ_42_ was incubated alone for 72 h, the turbidity increased rapidly (0.073 units). After co-incubation with CUR, CUR-CDs, and CUR-Fe_3_O_4_@CDs, the turbidity of the Aβ_42_ solution decreased, and the turbidity value of the CUR-Fe_3_O_4_@CDs intervention decreased significantly (0.025 units), indicating that CUR-Fe_3_O_4_@CDs have a significant inhibitory effect on the formation of Aβ_42_ fibrils, and its inhibitory effect is stronger than CUR and CUR-CDs (0.034 or 0.032 U, respectively).

CD measurement is one of the most important peptide spectroscopy methods for protein structural properties assessment. To determine the effect of CUR-Fe_3_O_4_@CDs on the conformational transition of Aβ_42_, we used CD spectrometry to observe the changes in secondary structures of Aβ_42_. The results are shown in Figure 3C. After Aβ_42_ was incubated for 6 days, a significant negative peak appeared at 200–220 nm, and the lowest value appeared at 218 nm, which was a characteristic peak of β-sheets (Schlenzig et al., 2009; Saraiva et al., 2010). After co-incubation with CUR, a significant negative peak still appeared at 218 nm, indicating that CUR did not significantly inhibit the transformation of Aβ_42_ fibrils secondary structure. However, with the addition of CUR-CDs and CUR-Fe_3_O_4_@CDs, the negative peak at 218 nm is significantly weakened, indicating that the Aβ_42_ fibrils gradually undergo a secondary structure transformation and restores the original Typical random coil conformation. The CD spectrum is consistent with the results of ThT analysis, which clearly confirms that CUR-Fe_3_O_4_@CDs can effectively inhibit the aggregation of Aβ fibrils.

Finally, we used TEM analysis to determine the inhibitory effect of CUR-Fe_3_O_4_@CDs on the ultrastructural properties of assembled Aβ aggregates (Jiang et al., 2019). The Aβ_42_ solution was incubated for 72 h to obtain abundant Aβ_42_ fibrils. The pre-formed Aβ_42_ fibrils were incubated with CUR, CUR-CDs, and CUR-Fe_3_O_4_@CDs for 72 h to prepare sample solutions. The results are shown in Figure 3D, the degree of aggregation of Aβ_42_ after 6 days incubation is obvious, and the fibrous network structure can be clearly seen. When CUR is incubated with Aβ_42_, fibrous aggregation still occurs, but the entanglement of Aβ_42_ fibrils is reduced to a certain extent. In the presence of CUR-CDs, most of Aβ_42_ forms a shorter Aβ fibrous structure, and a certain degree of aggregation occurs. It is worth noting that CUR-Fe_3_O_4_@CDs can induce the transformation of Aβ_42_ into small spherical particles or amorphous oligomers and exhibit a more significant inhibitory effect on the aggregation of Aβ polypeptides.

### Protection of PC12 Cells From the Toxicity of Aβ_42_ Fibrils

The ability of cells to uptake enough drugs is as important as their pharmacological activity. We use PC12 cells as a research model to study the internalization of CUR-Fe_3_O_4_@CDs in brain neurons. After 1 h of incubation, the cellular uptake of CUR-Fe_3_O_4_@CDs was evaluated by CLSM. As shown in Supplementary Figure 4, the fluorescence intensity of CUR (green) and CDs (blue) in PC12 cells was significantly high. This is of great significance for CDs to play a drug tracing role and curcumin to play a therapeutic role.

**FIGURE 4:**
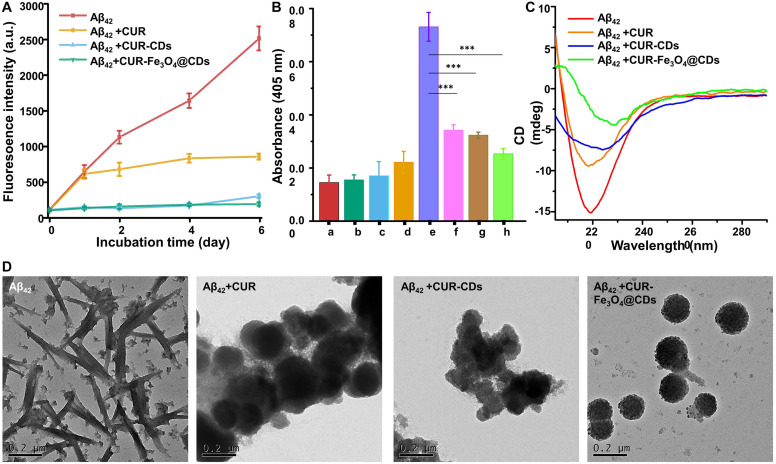
Fibril inhibition assay. **(A)** Monitoring Aβ_42_ fibril formation by ThT fluorescence assay in the presence of CUR, CUR-CDs, and CUR- Fe_3_O_4_@CDs (20 μM). **(B)** Turbidity of CUR (a), CUR-CDs (b), CUR-Fe_3_O_4_@CDs (c), Aβ_42_ monomer (d), Aβ_42_ fibrils (e), Aβ_42_ + CUR (f), Aβ_42_ + CUR-CDs (g), Aβ_42_ + CUR-Fe_3_O_4_@ CDs (h). **(C)** The CD spectra of Aβ_42_ fibrils after incubation for 72 h in the presence of CUR, CUR-CDs, and CUR-Fe_3_O_4_@CDs. **(D)** The TEM images of Aβ_42_ fibrils after incubation with CUR, CUR-CDs, and CUR-Fe_3_O_4_@CDs; (^∗^*P* < 0.05, ^∗∗^*P* < 0.01, ^∗∗∗^*P* < 0.001).

The ability of CUR-Fe_3_O_4_@CDs to inhibit Aβ_42_ aggregation suggests that it might be useful in blocking Aβ_42_-induced cellular toxicity. To address this question, MTT assays were used to investigate the cytotoxicity of Aβ_42_ fibrils in the absence and presence of the CUR-Fe_3_O_4_@CDs on PC12 cells. Aβ_42_ fibrils were prepared by incubating at 37°C for 6 days. The neurotoxicity of different concentration of Aβ_42_ fibrils is shown in Supplementary Figure 5. We found that after treating with 50 μM Aβ_42_ fibrils for 24 h, PC12 cells were observed to be significantly contracted, the protrusions were reduced, and the cell viability decreased to 47.17%. To understand the effect of CUR-Fe_3_O_4_@CDs on the cytotoxicity induced by Aβ_42_ fibrils, we pre-incubated Aβ_42_ fibrils with PC12 cells for 24 h and then added CUR-Fe_3_O_4_@CDs. The results are shown in Figure 4. After Aβ_42_ fibrils intervention, the cell survival rate dropped to 48.49%. Treatment of the PC12 cells with Aβ_42_ fibrils in the presence of 1 μg/mL CUR, CUR-CDs and CUR-Fe_3_O_4_@CDs significantly increased the survival of the cells to about 51.33, 53.57, and 87.47%, respectively. Aβ_42_ fibrils treated with 5 μg/mL CUR, CUR-CD, and CUR-Fe_3_O_4_@CDs increased the cell viability to 61.25, 62.18, and 94.78%, respectively. Taken together, these data demonstrate that CUR, CUR-CDs, and CUR-Fe_3_O_4_@CDs can reduce Aβ_42_ fibril toxicity. The data indicated that CUR-Fe_3_O_4_@CDs was the most effective.

In order to verify the cytotoxic effect of Aβ_42_ fibrils and the therapeutic potential of CUR-Fe_3_O_4_@CDs, we examined the morphological changes of PC12 cells caused by toxic fibrillation and the recovery of CUR-Fe_3_O_4_@CDs. As shown in Supplementary Figure 6, the cells in the control group were highly dense and had a spindle-shaped body with long dendrites and axons. When PC12 cells were treated with Aβ_42_ fibrils for 24 h, neurite loss, neurite contraction, cell body swelling, and overall destruction of the dendritic network were observed. However, when Aβ_42_ fibrils pre-intervened PC12 cells for 24 h and incubated with CUR-Fe_3_O_4_@CDs for 24 h, cell death decreased, and the surviving cells had significantly more normal morphology (Supplementary Figure 6). The results of cell morphology changes indicate that CUR-Fe_3_O_4_@CDs can reduce the cytotoxicity of Aβ_42_ fibrils. CUR-Fe_3_O_4_@CDs seems to be the most effective inhibitor of Aβ_42_ fibrils, which can enhance the viability of neuronal cells. These results are consistent with our previous experimental data.

### Reduce the Generation of ROS Induced by Aβ_42_ Fibrils in PC12 Cells

Aβ_42_ can cause many AD-like pathophysiological changes, of which nerve stress damage is the most important pathological damage (Schlenzig et al., 2009). Meanwhile, oxidative stress can change the metabolic process of APP, increase the expression and activity of β-secretase, and accelerate the production of Aβ_42_ (Saraiva et al., 2010). Due to the close relationship between oxidative stress and Aβ_42_ in the occurrence and development of AD, prevention and early treatment of AD can be carried out through the antioxidant pathway.

**FIGURE 5:**
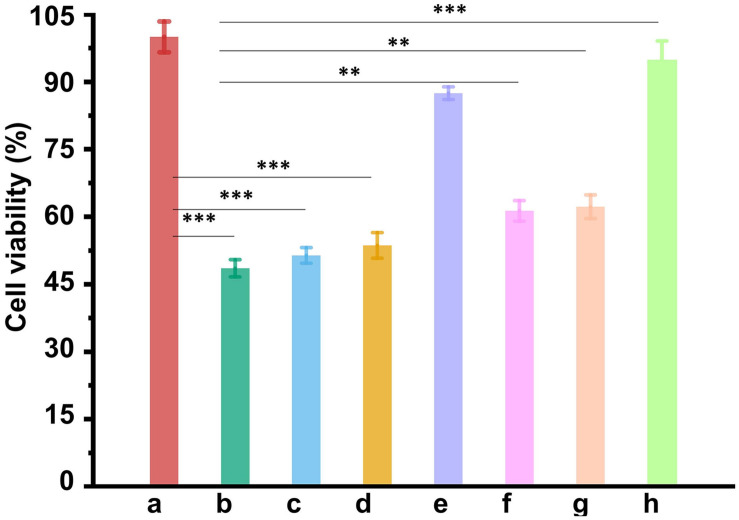
The ability of NPs to reduce Aβ_42_-mediated cytotoxicity. Cell viability of PC12 cells without treatment as control (a). Cell viability of PC12 cells treated with Aβ_42_ fibrils alone (b), Aβ_42_ fibrils + CUR (1 μg/mL) (c), Aβ_42_ fibrils + CUR-CDs (1 μg/mL of CUR) (d), Aβ_42_ fibrils + CUR-Fe3O4@CDs, (1 μg/mL of CUR) (e), Aβ_42_ fibrils + CUR (5 μg/mL) (f), Aβ_42_ fibrils + CUR-CDs (5 μg/mL of CUR) (g), and Aβ_42_ fibrils + CUR-Fe3O4@CDs (5 μg/mL of CUR) (h). ^∗^*p* < 0.05, ^∗∗^*p* < 0.01 and ^∗∗∗^*p* < 0.001.

To clarify any potential antioxidant effects of CUR-Fe_3_O_4_@CDs, DHE assay was used to measure the accumulation of ROS. DHE can enter the cell freely and is dehydrogenated to form ethidium bromide under the action of intracellular ROS. Ethidium bromide can combine with RNA or DNA to produce red fluorescence. When the level of ROS in the cell is higher, more ethidium bromide is produced, and the red fluorescence is stronger, and vice versa. In this way, DHE can be used to detect ROS levels. As shown in Figure 5, After treating PC12 cells with 50 μM Aβ_42_ fibrils for 24 h, the fluorescence intensity of ethidium bromide in the cells increased significantly. The presence of CUR and CUR-CDs caused the corresponding fluorescence intensity to be significantly reduced. Finally, the incubation of PC12 cells with CUR-Fe_3_O_4_@CDs showed that the fluorescence intensity decreased to close to the control level. These results clearly show that CUR-Fe_3_O_4_@CDs have a strong inhibitory effect on the production of ROS. The protective ability of CUR-Fe_3_O_4_@CDs is significantly greater than that of CUR and CUR-CDs, which is consistent with the experimental data of our previous study.

**FIGURE 6:**
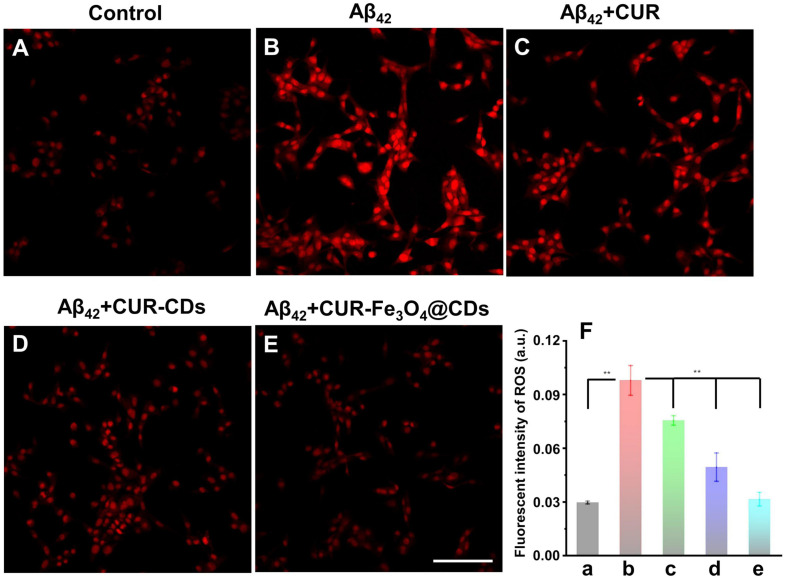
Detection of ROS production efficiency in PC12 cells. **(A)** The ROS production efficiency of control. **(B)** ROS production efficiency after treating with Aβ_42_ fibrils. **(C–E)** ROS production efficiency after treating with Aβ_42_ fibrils and CUR, CUR-CDs, and CUR-Fe_3_O_4_@CDs. **(F)** Quantitative analysis of the ROS level presented by the DHE fluorescence intensity, control (a), Aβ_42_ (b), β_42_ + CUR (c), Aβ_42_ + CUR-CDs (d), and Aβ_42_ + CUR-Fe_3_O_4_@CDs (e). Scale bar = 100 μm. (*p < 0.05, **p < 0.01 and ***p < 0.001).

## Conclusion

In summary, a new type of CUR delivery system has been successfully developed (CUR-Fe_3_O_4_@CDs). The results indicated that CUR-Fe_3_O_4_@CDs are a potential therapeutic candidate for Aβ fibril labeling and decomposition. Fluorescence analysis shows that CDs have inherent fluorescence characteristics, which can avoid the use of fluorescent labels. CUR-Fe_3_O_4_@CDs have good biocompatibility, which is non-toxic to PC12 cells and has a very low hemolysis rate. *In vitro* studies also show that CUR-Fe_3_O_4_@CDs have high specific affinity for Aβ_42_ and can significantly inhibit the aggregation of Aβ_42_ protein. More importantly, CUR-Fe_3_O_4_@CDs can rescue the PC12 cytotoxicity induced by Aβ_42_ fibrils and restore its cell morphology. The possible mechanism is related to the reduction of intracellular ROS production efficiency. We believe that the development of this new type of drug nanocarrier will provide useful tools for the suppression and elimination of amyloid and provide new ideas for the integration of imaging and AD treatment. Although these results are reliable and promising, more *in vitro* and *in vivo* experiments are still needed to further verify these results and better understand the anti-AD effect of CUR-Fe_3_O_4_@CDs.

## Data Availability Statement

The original contributions presented in the study are included in the article/Supplementary Material, further inquiries can be directed to the corresponding authors.

## Author Contributions

QH and XY designed the research. YK carried out the experiments. WZ, XL, YL, and MY performed the data analysis. JZ and MX participated in cell experiments. YK, QH, and XY wrote the manuscript. FL revised the manuscript. All authors checked the manuscript.

## Conflict of Interest

The authors declare that the research was conducted in the absence of any commercial or financial relationships that could be construed as a potential conflict of interest.
