# Supplementary material for: A Novel Nanosystem Realizing Curcumin Delivery Based on Fe3O4@Carbon Dots Nanocomposite for Alzheimer’s Disease Therapy
Source: Front Bioeng Biotechnol. 2020 Dec 3;8:614906. doi: 10.3389/fbioe.2020.614906 (PMC7744485; doi:10.3389/fbioe.2020.614906)
Supplement: Supplementary file 1 [file Data_Sheet_1.doc]

Supporting Information

**A novel nanosystem realizing curcumin delivery based on Fe_3_O_4_@carbon dots nanocomposite for** **Alzheimer’s disease therapy**

Ying Kuang^1,2^, Jingwen Zhang^2^, Mogao Xiong^2^, Weijia Zeng^2^, Xiaofeng Lin^2,3^, Xiaoqing Yi^2,*^, Yan Luo^2^, Min Yang^2^, Qitong Huang^2,*^

^1^ Guangdong Provincial Key Laboratory of Brain Function and Disease, Department of Neurobiology and Anatomy, Zhongshan School of Medicine, Sun Yat-sen University, Guangzhou 510080, PR China.

^2^ Oil-tea in Medical Health Care and Functional Product Development Engineering Research Center in Jiangxi, Key Laboratory of Biomaterials and Biofabrication in Tissue Engineering of Jiangxi Province, Key Laboratory of Prevention and Treatment of Cardiovascular and Cerebrovascular Diseases, Ministry of Education, Gannan Medical University, Ganzhou 341000, PR China.

^3^ Department of Chemistry, Shantou University, Guangdong 515063, PR China.

* Corresponding author: Xiaoqing Yi: **E-mail address:** keyi0115@126.com. **Tel:** +86-797-8169728.

Qitong Huang: **E-mail address:** hqt@gmu.edu.cn, hqtblue@163.com. **Tel:** +86-797-8169728.

**Figure S1**


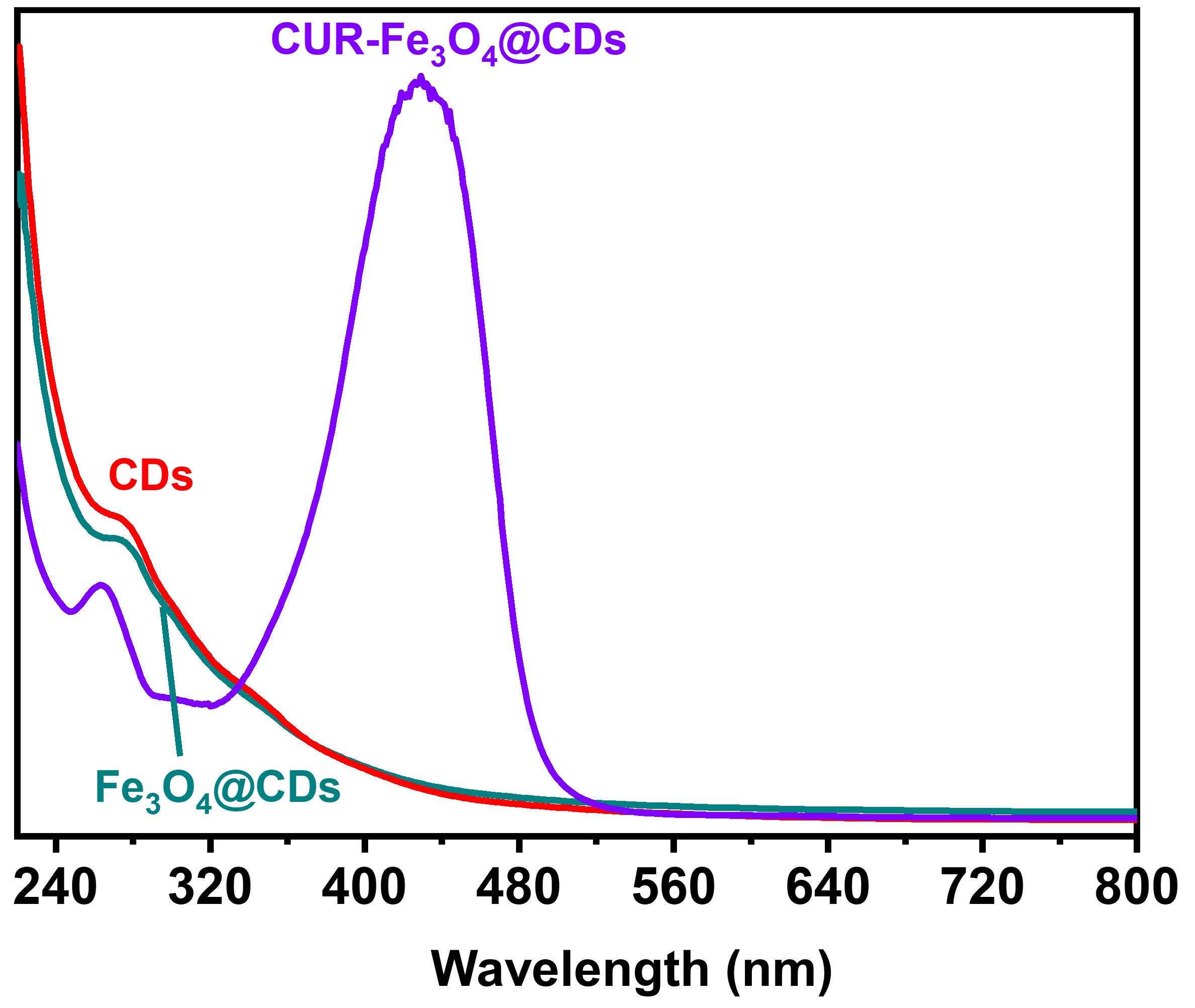


**FIGURE S1.** UV-vis absorbance of CDs, Fe_3_O_4_@CDs**,** and CUR-Fe_3_O_4_@CDs.

**Figure S2**


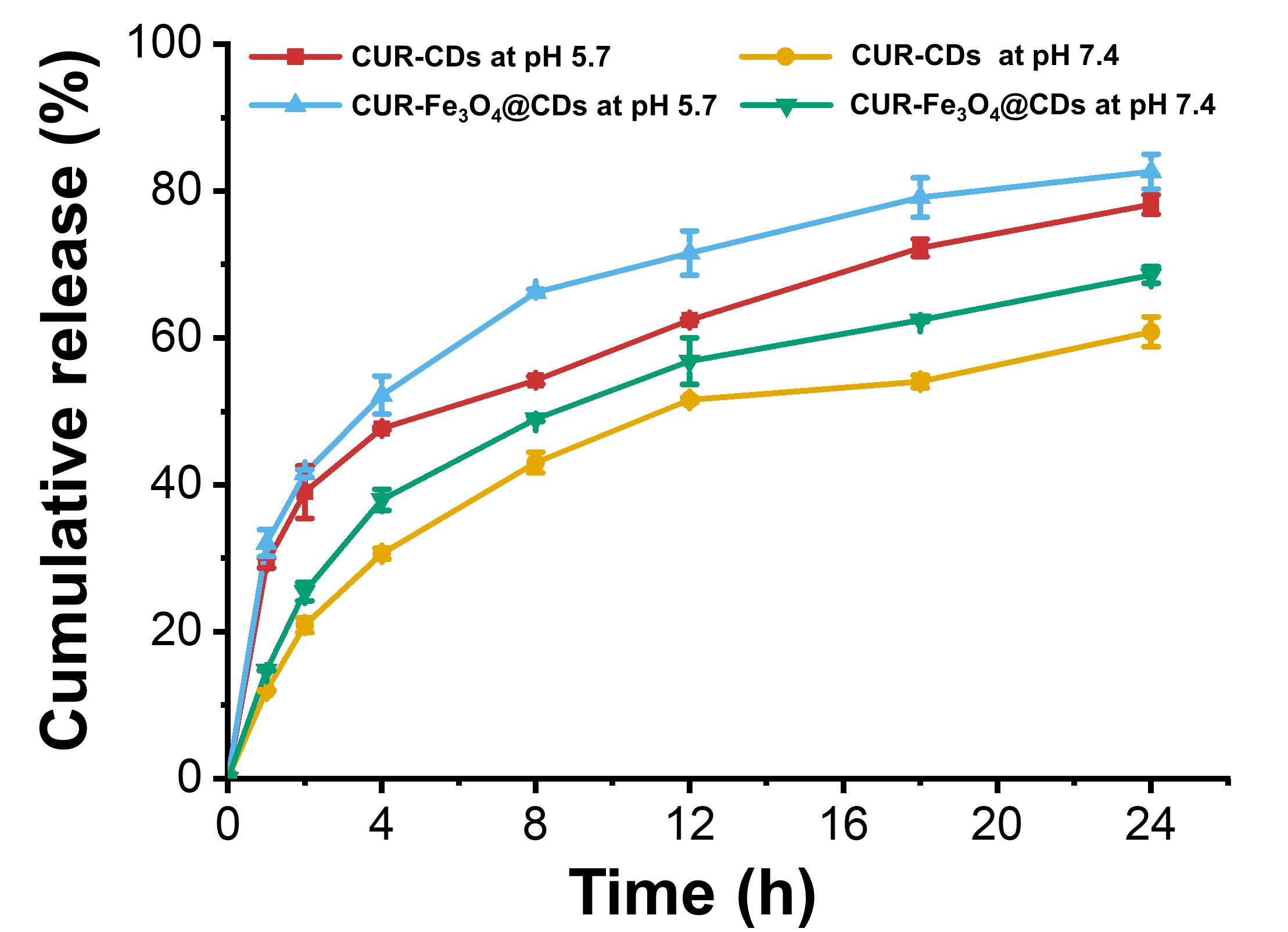


**FIGURE S2** In vitro release of CUR from CUR-CDs and CUR-Fe_3_O_4_@CDs at different pH in PBS.

**Figure S3**


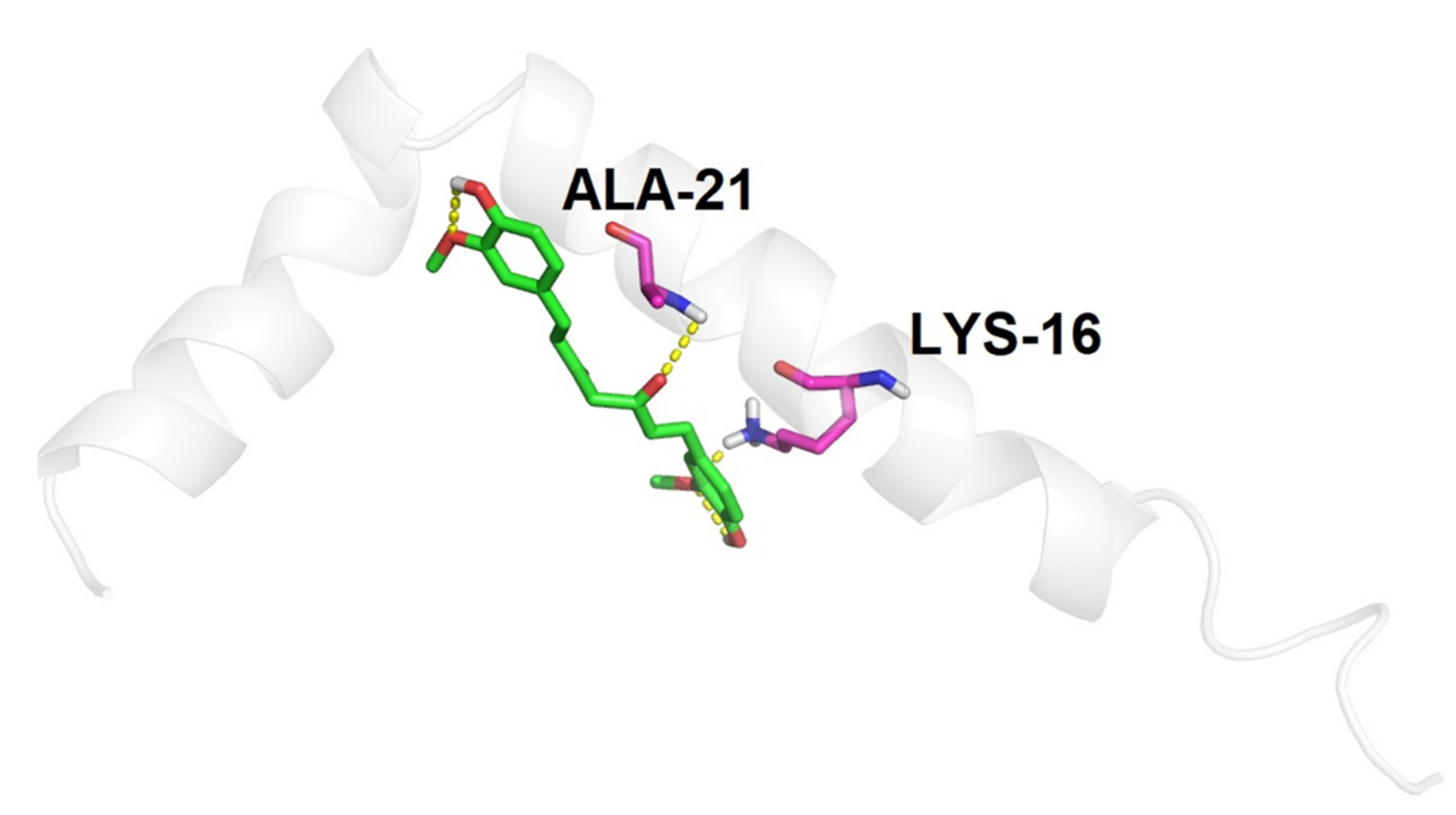


**FIGURE S3** Docking calculation of CUR and Aβ_42_.

**Figure S4**


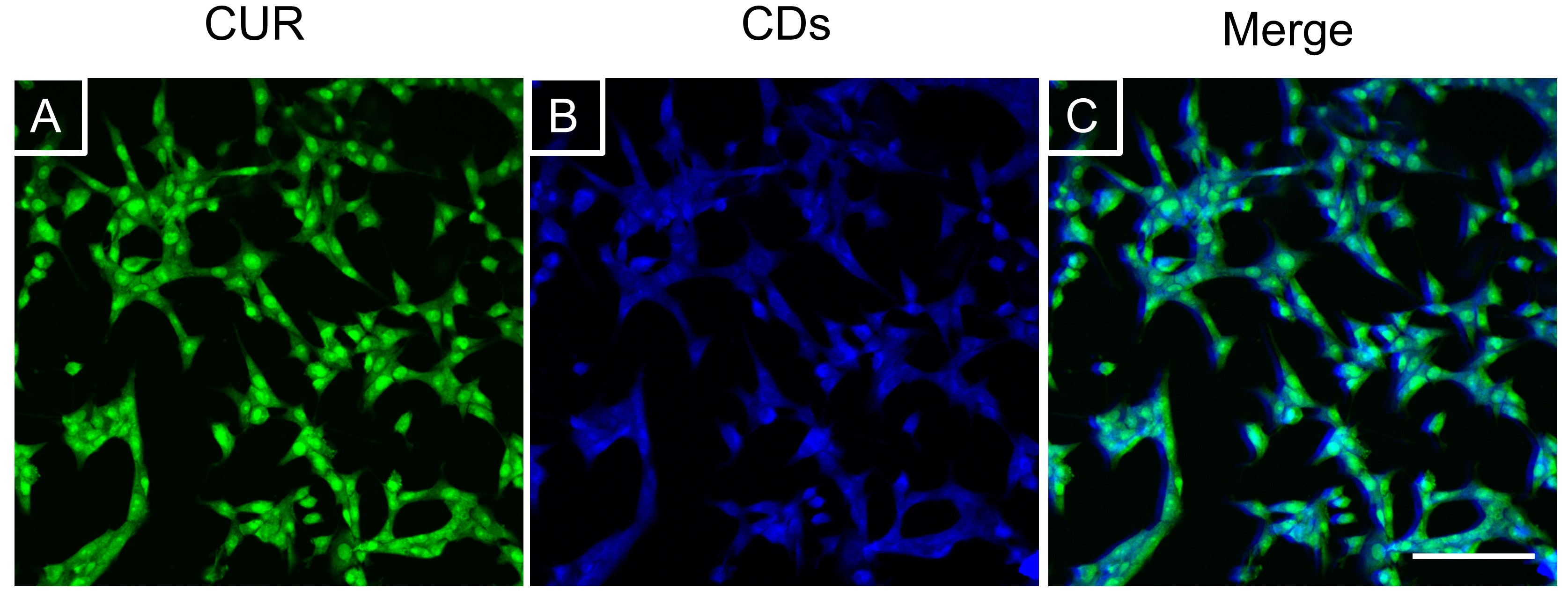


**FIGURE S4** Fluorescence image after incubation of PC12 cells with CUR-Fe_3_O_4_@CDs for 1 h. Scale bar=100 μm.

**Figure S5**


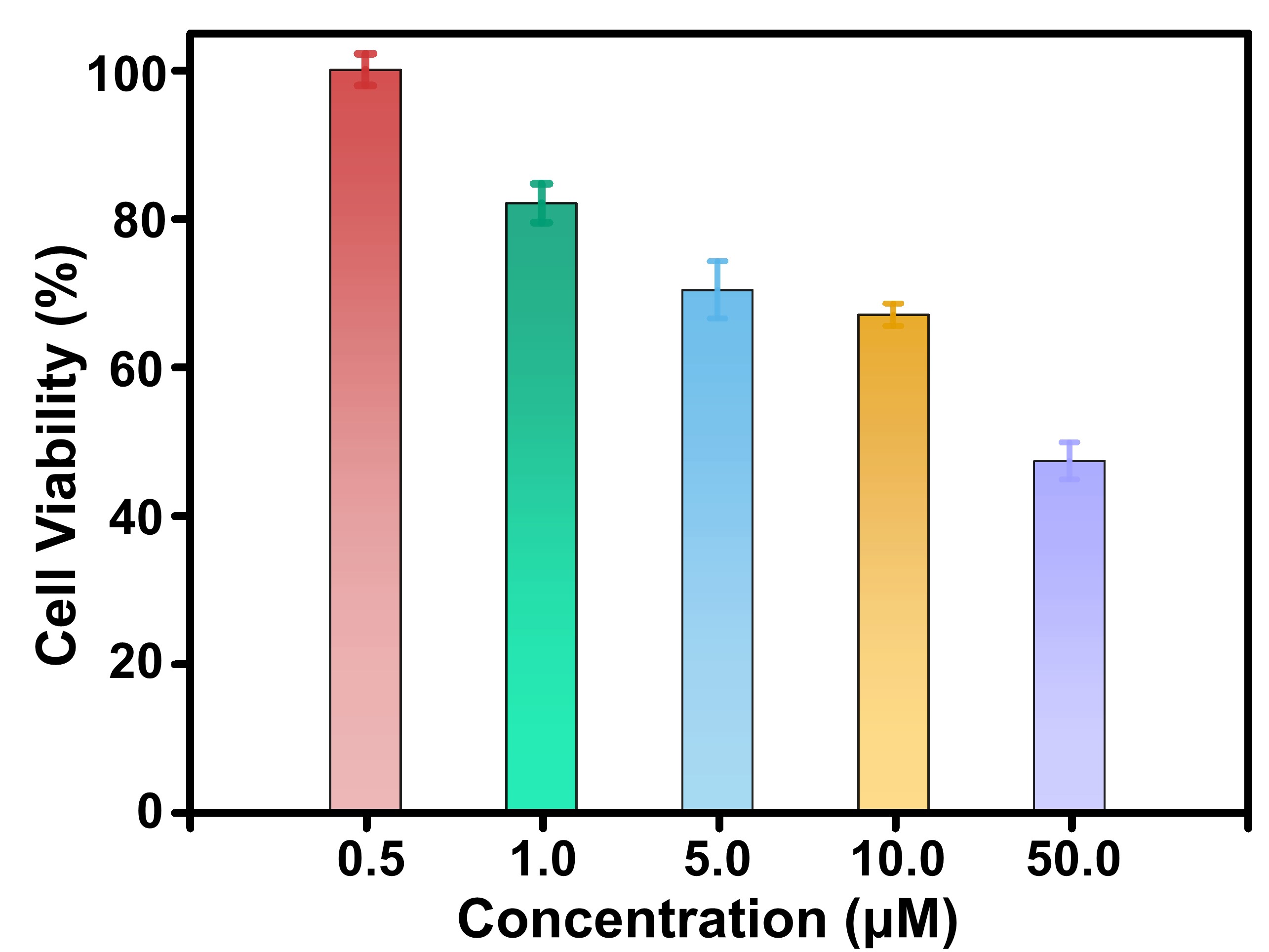


**FIGURE S5** PC12 cell viabilities after incubation with different concentrations of Aβ_42_ fibrils for 24 h.

**Figure S6**


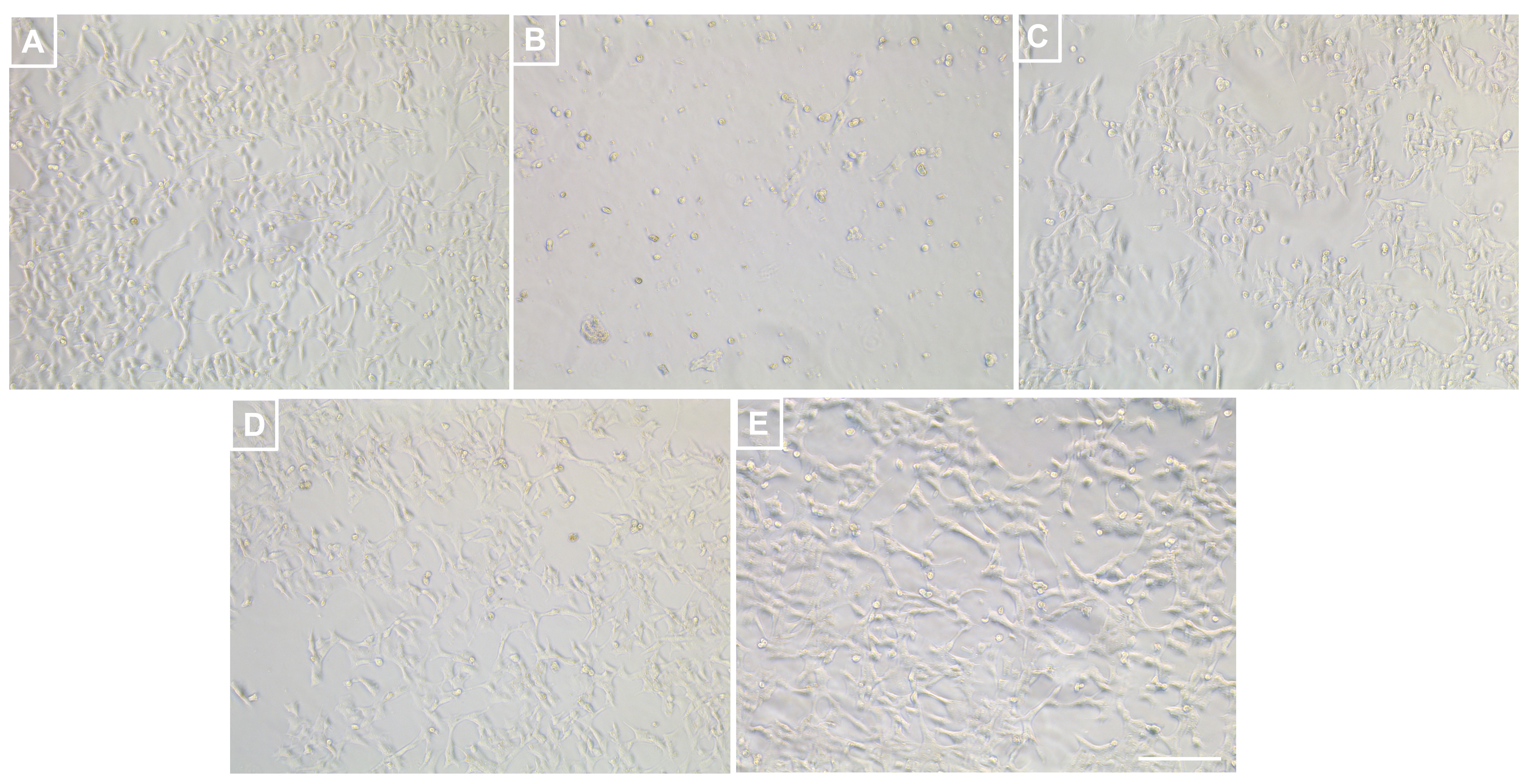


**FIGURE S6** Morphological changes of PC12 cells. (**A**) Under control. (**B**) Treated with Aβ_42_ fibrils for 24 h. (**C**) Treated with Aβ_42_ fibrils for 24 h, then treated with CUR for 24 h. (**D**) Treatment with Aβ_42_ fibrils for 24 h, followed by treatment with CUR-CDs for 24 h. (**E**) Treatment with Aβ_42_ fibrils for 24 h, followed by treatment with CUR-Fe_3_O_4_@CDs for 24 h. Scale bar= 100 μm.
